# A Few Practical Observations on Diet, Especially That of Children and Invalids, with Inquiry into the Effects of Alcohol on the System

**Published:** 1851-04

**Authors:** 


*A few Practical Observations on Diet, especially that of Children and Invalids, with Inquiry into the Effects of Alcohol on the System.* By Charles Beckett, M. R. C. S. London: pamphlet, pp. 17.

This little treatise is much too brief. Upon a subject of so much importance, we could have wished Mr. Beckett to have extended his inquiries, and produced a work worthy of his talents. We extract from it the following passages:

"It evidently never was intended that children which have not cut their teeth, should be furnished with food requiring mastication. Yet how constantly do we see them supplied with portions of meat, pastry, and other, (to them,) indigestible substances, in direct violation of this plain law. The result is, that instead of premature development of strength, we have irritation of the lining membrane of the stomach and bowels, which induces diarrhea, and may result in the more formidable evils of convulsions or atrophy. The difficulty of getting parents to attend to simplicity of diet in children under five years of age, is a fertile source of disappointment to the practitioner, as well as of atrophy, marasmus and infantile remittent fever, all fatal forms of disease to the child. This foolish system of indulgence extends to the lowest ranks of society, and seems often to be the most practiced where it is likely to be the most injurious."—pp. 6, 7.

"In the management of infants, it is as cruel as it is unwise, to depend *mainly* upon aliments which are chemically incapable of affording adequate nutrition. Sago, for instance, as an article of diet for children, is, *where relied upon,* most objectionable. I have seen several cases of the unhealthy fattening of children, followed by extreme emaciation, difficult dentition, and even where worse consequences have apparently arisen from this cause. We shall find sago to be one of those compounds incapable of organization, from its chemical constitution; and, moreover, being deficient in salts of lime, it will not afford the requisite supply for the growth of the bones, and development of the teeth—thus directly tending to induce rickets and tardy dentition. No wonder, then, that it fails to supply the place of homelier food." J. R.

